# Depression among nursing students in Pakistan: a systematic review and meta-analysis

**DOI:** 10.1186/s40359-026-04385-w

**Published:** 2026-04-14

**Authors:** Hamideh Ebrahimi, Masoud Mohammadnezhad, Ghazala Irshad

**Affiliations:** 1 Lahore School of Nursing, The University of Lahore (UoL), Lahore, Pakistan; 2 School of Nursing and Midwifery, Birmingham City University, Birmingham, UK; 3 Department of Public Health, Daffodil International University (DIU) Daffodil Smart City (DSC), Savar, Dhaka 1216 Bangladesh; 4 University College of Medicine and Dentistry (UCMD), The University of Lahore, Lahore, Pakistan

**Keywords:** Depression, Prevalence, Nursing students, Pakistan, Systematic Review

## Abstract

**Background:**

Depression is a common psychological disorder that adversely affects emotional functioning, motivation, and academic performance. Nursing students are at increased risk due to academic load, clinical responsibilities, and frequent exposure to patient suffering. In Pakistan, cultural expectations, financial pressure, and stigma surrounding mental health may further exacerbate vulnerability. This study aimed to synthesize existing evidence on the prevalence of depression among nursing students in Pakistan.

**Methods:**

This systematic review and meta-analysis was conducted in accordance with the PRISMA 2020 guidelines and registered in the PROSPERO database (CRD420251178412). A comprehensive literature search was conducted across MEDLINE (PubMed), Embase, Scopus, Web of Science, APA PsycINFO, and Pakistani academic databases without year restrictions. Studies reporting the prevalence of depression among nursing students using validated tools (e.g., DASS-21, BDI-I, BDI-II, SSDS) were included. Data extraction and quality appraisal were performed independently by two reviewers using the Joanna Briggs Institute (JBI) tool. A random-effects model was applied using STATA version 17. Heterogeneity was assessed using the Cochran Q test and I² statistic. Subgroup analyses and meta-regression explored potential sources of variability.

**Results:**

Eleven studies comprising 2,253 nursing students met the eligibility criteria. The pooled prevalence of depression was 37% (95% CI: 25%–50%), with high heterogeneity across studies (I² > 95%). Subgroup analyses showed no significant differences by measurement tool or province. Meta-regression indicated no significant association between year of publication and reported prevalence.

**Conclusion:**

Depression is a notable mental health concern among nursing students in Pakistan. Strengthening psychological support services and implementing stress management interventions within nursing education are essential. Further high-quality, multi-center, and longitudinal research is needed.

**Supplementary Information:**

The online version contains supplementary material available at 10.1186/s40359-026-04385-w.

## Introduction

Depression is a common psychological disorder that affects individuals globally, leading to decreased energy, motivation, concentration, and functioning. It impacts emotions, thoughts, and actions, ultimately reducing quality of life [1, 2]. The findings of this global systematic review indicate a substantial increase in the prevalence of depressive symptoms among adolescents over the past two decades. At the global level, evidence from a systematic review and meta-analysis demonstrated a substantial increase in the prevalence of depressive symptoms among adolescents worldwide over the past two decades, with an approximate 14% rise when comparing the periods 2001–2010 and 2011–2020 [3]. People with depression frequently face ongoing fatigue, feelings of hopelessness, decreased productivity, and challenges in completing everyday tasks. Together, these issues can significantly impact a person’s capacity to manage life’s difficulties and stressful situations [3, 4].

During university years, individuals experience a crucial phase in their lives as they transition from adolescence to adulthood. This period brings increased responsibilities, academic challenges, social adjustments, and often physical distance from family, all of which can impact students mental well-being [5].

Nursing students face unique and heightened challenges compared to other university students [6] In addition to the typical academic pressures, they also deal with practical training in hospital settings, direct exposure to patients’ suffering, and the responsibility of balancing personal and academic life. These factors can significantly increase stress levels among nursing students, putting them at a greater risk of psychological issues such as anxiety, burnout, and depression [7, 8].

In Pakistan, nursing students face a higher risk of stress due to specific cultural norms related to both the nursing profession and mental health, as well as economic challenges and structural characteristics of the educational system. Culturally, nursing is often perceived as a low-status profession compared to medicine, and in some contexts, female nursing students may face gender-based expectations, limited professional autonomy, and family pressure regarding career choice. In addition, mental health problems are frequently stigmatized, and emotional distress may be viewed as a sign of personal weakness, discouraging students from seeking psychological support. Factors such as financial strain, societal expectations, and stigma surrounding mental health can therefore exacerbate psychological stress [9]. Reluctance to seek help due to fear of social judgment may further negatively affect students’ mental health and clinical performance [10]. Depression can also impair patient care by reducing communication quality, empathy, and clinical accuracy [11].

While numerous studies have investigated depression among nursing students in Pakistan, the existing evidence remains fragmented, and the use of different depression assessment instruments has resulted in heterogeneous and sometimes conflicting findings. Moreover, although several international systematic reviews and meta-analyses have reported the prevalence of depression among nursing students worldwide, these studies often combine data from diverse educational systems, cultural contexts, and socioeconomic settings, which may obscure important country-specific determinants of mental health [6, 12].Therefore, a systematic review and meta-analysis focusing specifically on Pakistan is warranted to synthesize the available evidence, provide a more accurate and context-specific pooled estimate of depression prevalence, and support the development of targeted interventions. Accordingly, the aim of this study was to conduct a systematic review and meta-analysis to estimate the prevalence of depression among nursing students in Pakistan and to explore heterogeneity in prevalence estimates based on assessment instruments and geographical regions, in order to inform nursing education policies and the development of student mental health support services.

## Method

This systematic review and meta-analysis followed the Preferred Reporting Items for Systematic Reviews and Meta-Analyses (PRISMA 2020) guidelines [13] and was registered in the PROSPERO database under the registration number CRD420251178412.

### Search strategy

The search was performed across major international databases, including MEDLINE (via PubMed), Web of Science Core Collection, Embase, Scopus, and APA PsycINFO. Additionally, manual searches of Pakistani academic resources such as Pakistan Journal of Medical Sciences, Journal of the Pakistan Medical Association, and PakMedi Net were conducted. The final search was completed on 10 October 2025. The search strategy utilized a combination of controlled vocabulary (MeSH/ Emtree) and free-text terms related to depression, nursing students, and nursing education in Pakistan. Boolean operators AND and OR were applied to combine search terms and capture all relevant studies. No publication year limit was applied to maximize the comprehensiveness of retrieval. A detailed search syntax for each database is provided in Supplementary File 1.

The PEO framework was used to determine the eligibility criteria and guide the formulation of the research question:

- Population (P): Nursing students enrolled in diploma, undergraduate, or postgraduate nursing programs in Pakistan.
- Exposure (E): The exposure of interest was being a nursing student enrolled in diploma, undergraduate, or postgraduate nursing programs, as this population is exposed to academic, clinical, and psychosocial stressors associated with nursing education.
- Outcome (O): The prevalence and severity of depression.

### Inclusion and exclusion criteria

Studies were included if they focused on nursing students enrolled in diploma, undergraduate, or postgraduate nursing programs in Pakistan and reported the prevalence of depression or provided sufficient data to calculate it. Depression had to be assessed using validated screening or diagnostic instruments, such as the PHQ-9, BDI, DASS-21, HADS, CES-D, or other standardized tools. Only articles published in English or Urdu were considered. Furthermore, the review included cross-sectional or other observational studies that provided quantitative data on the prevalence or severity of depression among nursing students. The literature search was conducted up to 10 October 2025.

Studies that did not provide separate data for nursing students, or that used instruments combining depression with other psychological constructs such as anxiety or stress (e.g., the Aga Khan Stress and Depression Scale), were excluded, as they did not allow for independent estimation of depression prevalence. This exclusion criterion represents a minor deviation from the registered PROSPERO protocol and was implemented after full-text screening to enhance methodological consistency and comparability across studies.

In addition, narrative reviews, systematic reviews, meta-analyses, letters to the editor, commentaries, case reports, and studies without original quantitative data were excluded. Studies that did not provide sufficient information to calculate depression prevalence or mean scores were also excluded. Unpublished studies and articles that could not be accessed, even after contacting the corresponding author, were not considered.

### Study selection

**Fig. 1:**
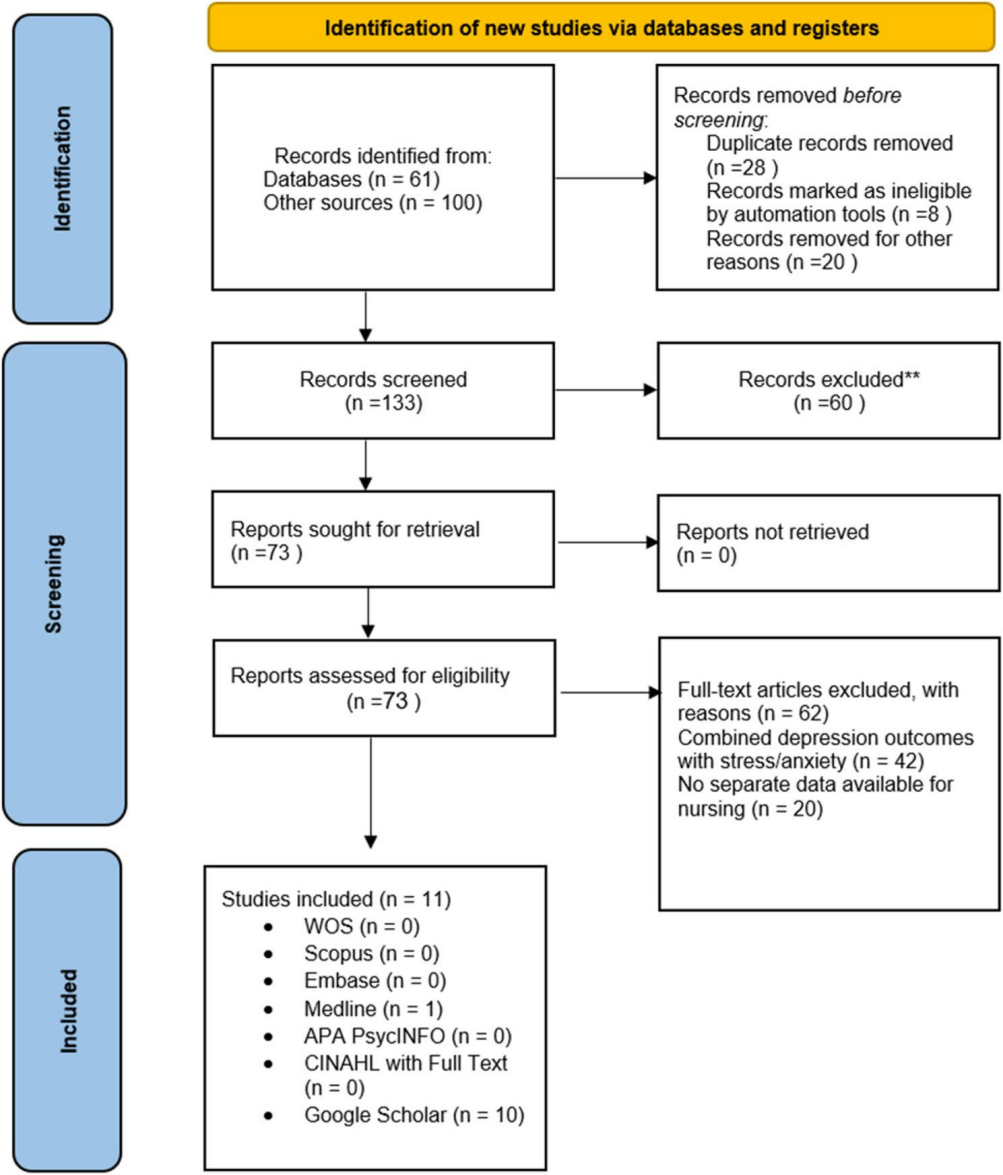
PRISMA flow diagram

All search results were imported into EndNote software, and duplicate records were removed automatically. The study screening was conducted in two sequential phases. In the first phase, two reviewers independently examined the titles and abstracts of all retrieved records to assess their relevance to the research question. In the second phase, the full texts of studies that met the initial eligibility criteria were reviewed in detail to determine final inclusion. Any disagreements between the reviewers were resolved through discussion, and when necessary, by consulting a third reviewer to reach consensus. Although the registered PROSPERO protocol was largely followed, a minor amendment was implemented after full-text screening and data extraction. Specifically, studies using instruments that combined depression and stress scores, such as the Aga Khan Stress and Depression Scale, were excluded because they did not allow for an independent estimation of depression prevalence. This amendment was made to enhance methodological consistency and the validity of the pooled estimates. The overall study selection process is illustrated in the PRISMA flow diagram (Fig. 1).

### Data extraction

Two reviewers independently extracted data from all studies that met the inclusion criteria. The extracted information included: first author’s name, year of publication, study setting and geographical region, total sample size, sample characteristics (e.g., gender distribution, Mean age, ), depression assessment instrument used, cut-off score applied, and the reported prevalence or mean score of depression. Where available, data on the severity levels of depression (e.g., mild, moderate, severe) and study quality/assessment scores were also recorded, and the quality of the included studies was assessed using the Joanna Briggs Institute (JBI) critical appraisal tools [14]. Any disagreements during the data extraction process were resolved through discussion and mutual consensus.

### Measurement tools for depression

In this systematic review, only three valid tools were identified in the included studies: the DASS-21, BDI-I, and BDI-II.

The DASS-21 comprises 21 items, categorized into three subscales for depression, anxiety, and stress. For this study, only the depression subscale (7 items) was utilized. Each item is rated on a scale from 0 to 3, and the total score is doubled to align with the 42-item version. In accordance with established guidelines, a cut-off score of ≥ 10 on the depression subscale (after score multiplication) was used to indicate the presence of depressive symptoms [15]. The BDI-I consists of 21 items with scores ranging from 0 to 3 for each item, assessing the severity of emotional-behavioral symptoms of depression [16]. The BDI-II is a revised version based on DSM-IV criteria, comprising 21 items scored from 0 to 3, evaluating symptoms over the past two weeks. In this review, a cut-off score of ≥ 14 was used to define depressive symptoms, corresponding to the threshold for mild depression .To ensure consistency and comparability of findings across studies, the recommended cut-off points for each tool were applied to determine prevalence. Scores equal to or exceeding the threshold for “mild depression” were categorized as indicative of depression [17].

One study included in this review utilized the Siddiqui–Shah Depression Scale (SSDS) to evaluate depression. This self-report questionnaire was specifically developed and culturally adapted in Pakistan by Siddiqui and Shah to assess the severity of depressive symptoms in adult and adolescent populations. The SSDS comprises 52 items, each rated on a five-point Likert scale (ranging from 0 = never to 4 = very much). Based on the developers’ recommendations, a cut-off score of ≥ 37 was used to indicate depressive symptoms, and this threshold was applied to estimate prevalence in the included study This tool holds significance for its cultural and linguistic alignment with the Pakistani population, and its validity and reliability have been validated in numerous studies [18]. Table 1 provides a summary of the measurement tools used in the included studies, including the number of items, scoring systems, and applied cut-off points, to facilitate clearer understanding and comparison across instruments.

**Table 1:** Summary of the measurement tool

Measurement Tool	Number of Items	Scoring System	Cut-off Point
DASS-21 (Depression subscale)	7 (of 21)	Items scored 0–3; total score multiplied by 2	≥ 10
Beck Depression Inventory (BDI-I)	21	Items scored 0–3	≥ 14
Beck Depression Inventory-II (BDI-II)	21	Items scored 0–3	≥ 14
Siddiqui–Shah Depression Scale (SSDS)	52	Items scored 0–4 (Likert scale)	≥ 37

### Quality assessment

The methodological quality of the included studies was assessed using the Joanna Briggs Institute (JBI) critical appraisal checklist for prevalence studies. This checklist consists of nine methodological items addressing key domains such as sample selection, sample size adequacy, validity of measurement tools, and appropriateness of statistical analysis. Each item was rated as “Yes,” “No,” “Unclear,” or “Not applicable.” A score of 1 was assigned for each “Yes” response, while all other responses were scored as 0.

As all nine items were applicable to the included studies, the total quality score for each study ranged from 0 to 9. Based on the total score, studies were categorized into three quality levels: low quality (scores ≤ 3), moderate quality (scores 4–6), and high quality (scores ≥ 7).

Overall, most studies were of moderate methodological quality. The most commonly identified methodological limitations included the use of non-probability sampling methods, insufficient reporting of response rates, and limited information regarding strategies to address potential confounding factors. These issues should be considered when interpreting the pooled prevalence estimates [14].

### Data analysis

Statistical analyses were conducted using STATA version 17. Heterogeneity among studies was assessed using the Cochran Q test and the I² statistic, with conventional thresholds applied to interpret heterogeneity as low (< 25%), moderate (25–50%), substantial (50–75%), or very high (> 75%). Given the presence of substantial heterogeneity, a random-effects model using the DerSimonian–Laird estimator was applied, as this approach accounts for both within-study and between-study variability and is commonly used in meta-analyses involving heterogeneous populations. The pooled prevalence of depression was estimated and presented using forest plots with 95% confidence intervals. To enhance consistency and comparability across studies, prevalence estimates were recalculated based on standardized cut-off points for each assessment instrument (DASS-21, BDI-I, and BDI-II), with scores at or above the threshold for mild depression classified as indicative of depressive symptoms. Subgroup analyses were performed according to depression measurement tools and geographic regions to explore potential sources of heterogeneity. Meta-regression analyses were conducted to examine the effects of publication year and sample size on reported depression prevalence. Publication bias was assessed using visual inspection of funnel plots and Egger’s regression test, with adjustment using the trim-and-fill method when evidence of asymmetry was detected. Given the extremely high heterogeneity observed across studies, the pooled prevalence estimate should be interpreted with caution, as it represents an approximate summary across heterogeneous populations, settings, and measurement tools rather than a precise national prevalence.

## Results

### Characteristics of included studies

The majority of the studies (72.7%) were conducted in the province of Sindh, while the remaining studies (27.3%) were conducted in Punjab. No eligible studies were identified from other major regions of the country, including Khyber Pakhtunkhwa and Balochistan, highlighting a substantial geographical gap in the existing literature. The DASS-21 was the most commonly used measurement instrument to assess depression, appearing in 4 studies (*n* = 4, 36.4%). The Beck Depression Inventory (BDI) and the Beck Depression Inventory-II (BDI-II) were each used in 3 studies (*n* = 3, 27.3%). In contrast, the Siddiqui–Shah Depression Scale (SSDS) was utilized in only one study (*n* = 1, 9.1%). The characteristics of the studies are summarized in Table 2. The methodological quality assessment of the included studies using the Joanna Briggs Institute (JBI) critical appraisal tool indicated that most studies were of moderate methodological quality (9 out of 11 studies), while 2 studies were rated as high quality. The most frequently identified methodological concerns were related to sample size adequacy and the lack of clear reporting of response rates or participant attrition during questionnaire administration. In several studies, although sample sizes were reported, insufficient justification was provided regarding whether the sample size was adequate to generate precise prevalence estimates. Furthermore, many studies did not clearly report the number of questionnaires distributed, the number returned, or the extent of participant non-response or attrition. These limitations primarily corresponded to JBI checklist items addressing sample size adequacy and response rate management and should be considered when interpreting the pooled prevalence estimates.

**Table 2:** Characteristics of the Included Studies

Author	Year	Location	Mean	Target group	SD	Sample Size	Prevalence	Male	Female	Quality Of Study	Tool	Cut of Point
Awan [9]	2025	Sindh	-	N.S	-	170	30.6	50	120	High	DASS-21	≥ 10
Kumar [19]	2025	Sindh	-	N.S	-	110	26	66	44	Moderate	DASS-21	Mild (≥ 10
Shah Hussain [20]	2025	Sindh	-	N.S	-	320	60.6			Moderate	DASS-21	DASS-21 ≥ 14
Mushtaq [21]	2024	Sindh	21.41	N.S	2.25	555	20.2	-	555	Moderate	SSDS	SSDS ≥ 37
Khalid Hussain [22]	2024	Sindh	-	N.S	-	120	44.2	69	51	Moderate	BDI	≥ 21
Ali [23]	2024	Sindh	23.2	N.S	4.2	223	9.86	110	113	High	DASS-21	≥ 10
Manzoor [24]	2024	Punjab	21.57	N.S	1.16	149	34.9	-	149	Moderate	BDI-II	Normal (0–10), Mild (11–16), Borderline (17–20), Moderate (21–30), Severe (31–40), Extreme (≥ 41)
Khalid Hussain, Sultan Muhammad [25]	2024	Sindh	-	N.S	-	120	44.2	69	51	Moderate	BDI	≥ 21
Khawar [26]	2022	Punjab	-	N/S	-	187	49.2	-	-	Moderate	BDI-II	BDI-II ≥ 20
Fatima [27]	2019	Punjab	21.57	N/S	1.16	149	14.1	-	-	Moderate	BDI	≥ 17
Khan [28]	2019	Sindh	29.4	N/S	5.6	150	76	72	78	Moderate	BDI-II	≥ 14

### Meta-analysis

In this meta-analysis, the prevalence of depression among nursing students in Pakistan was calculated using a random-effects model with the DerSimonian–Laird estimator. The overall prevalence was 37% (95% CI: 0.25 to 0.50), with a statistically significant z-value of 5.76 (*p* < 0.001). Heterogeneity across studies was substantial, with Cochran’s Q = 509.39 (df = 10, *p* < 0.001) and an I² value of 98%. The between-study variance was estimated at Tau² = 0.0446. Individual study prevalence estimates ranged from 0.10 to 0.76, with study weights in the pooled model ranging from 8.93% to 9.28%, indicating a balanced contribution of each study to the final effect size.

**Fig. 2:**
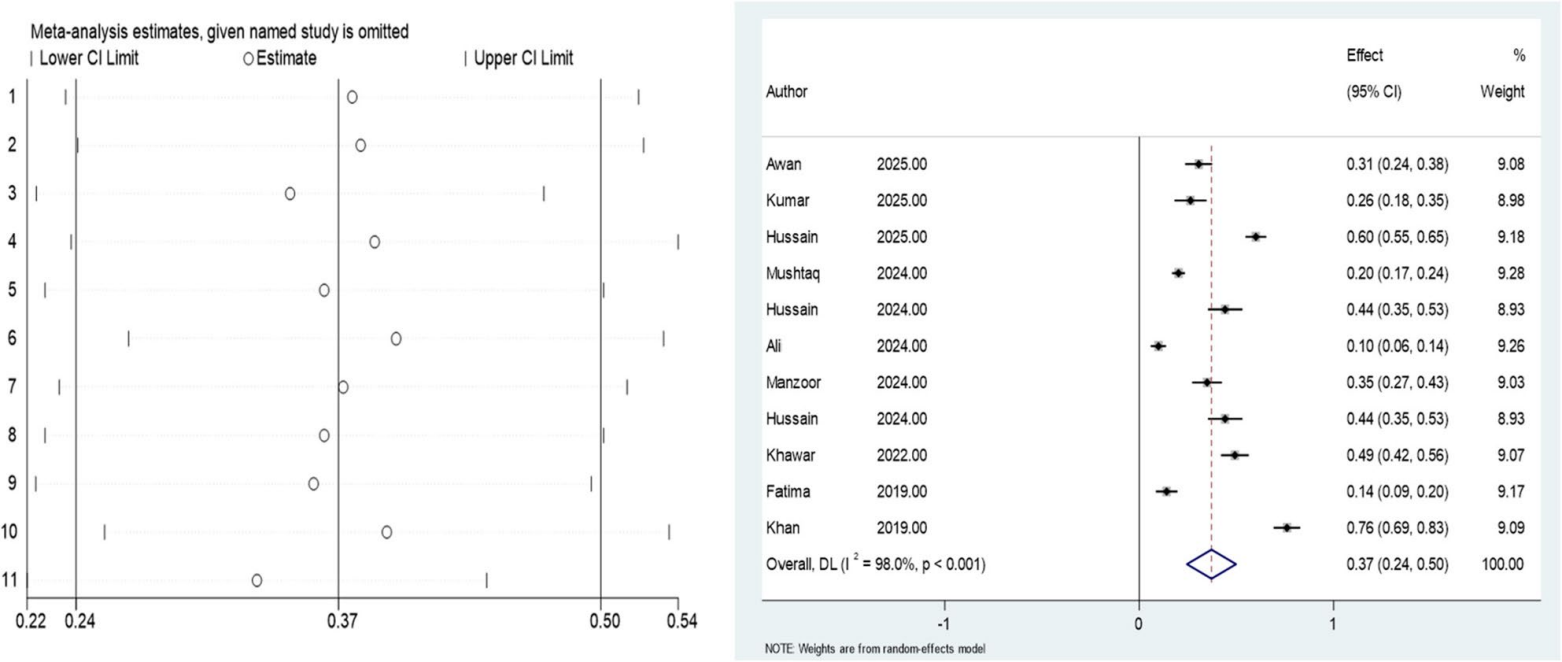
Forest plot and leave-one-out sensitivity analysis for the pooled prevalence of depression among nursing students in Pakistan

A leave-one-out sensitivity analysis was conducted to assess the robustness of the pooled prevalence estimate. The results showed that the pooled prevalence estimates ranged from 0.33 to 0.40 when each study was excluded one at a time, compared to the overall pooled estimate 37(95% CI: 0.24 to 0.50) when no studies were excluded. The removal of any single study did not significantly alter the pooled estimate, indicating the stability of the results. Figure 2 displays the forest plot of individual and pooled prevalence estimates, as well as the leave-one-out sensitivity analysis, demonstrating the stability of the pooled estimate when each study was sequentially excluded. Notably, only two included studies were rated as high quality according to the JBI checklist, while the remaining studies were of moderate quality. The leave-one-out sensitivity analysis demonstrated that exclusion of individual studies, including those of moderate methodological quality, did not materially change the pooled prevalence estimate, indicating that the overall findings were robust and not driven by any single study.

### Subgroup analysis based on assessment instruments

**Fig. 3:**
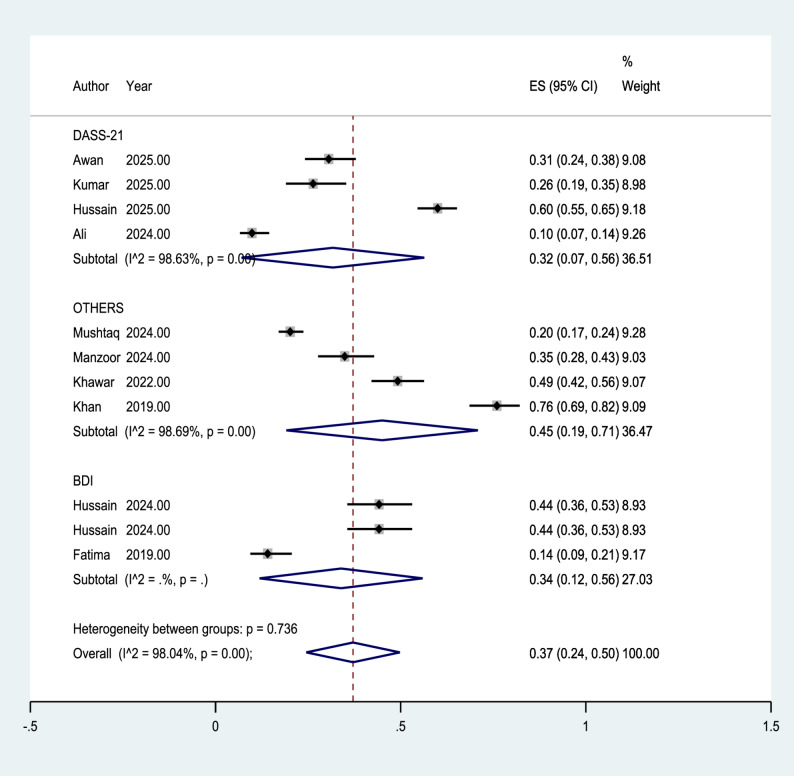
Forest plot of studies stratified by depression assessment instruments

In the subgroup analysis conducted according to the depression assessment instruments, the included studies were divided into three groups: DASS-21, BDI, and other instruments. The combined prevalence of depression among studies using the DASS-21 scale was 0.32 (95% CI: 0.07 to 0.56). For studies using the BDI, the combined prevalence was 0.34 (95% CI: 0.12 to 0.56). In the subgroup categorized as other instruments, the combined prevalence was 0.45 (95% CI: 0.19 to 0.71). The test for subgroup differences showed that the variation in combined prevalence across the different assessment tools was not statistically significant.Therefore, while the estimated prevalence varied numerically among instrument types, these differences were not statistically significant. Within each instrument category, there was substantial within-group heterogeneity observed (I² > 98%), indicating significant variability in prevalence estimates among studies. Figure 3 displays the subgroup forest plot of the included studies based on the depression assessment instruments.

### Subgroup analysis based on province

A subgroup analysis was conducted to explore potential differences in the prevalence of depression among nursing students in different provinces of Pakistan, specifically Punjab and Sindh. The combined prevalence estimate for studies in Punjab was 0.326 (95% CI: 0.112 to 0.540) using the random-effects model. In contrast, studies in Sindh showed a combined prevalence of 0.388 (95% CI: 0.227 to 0.550).

**Fig. 4:**
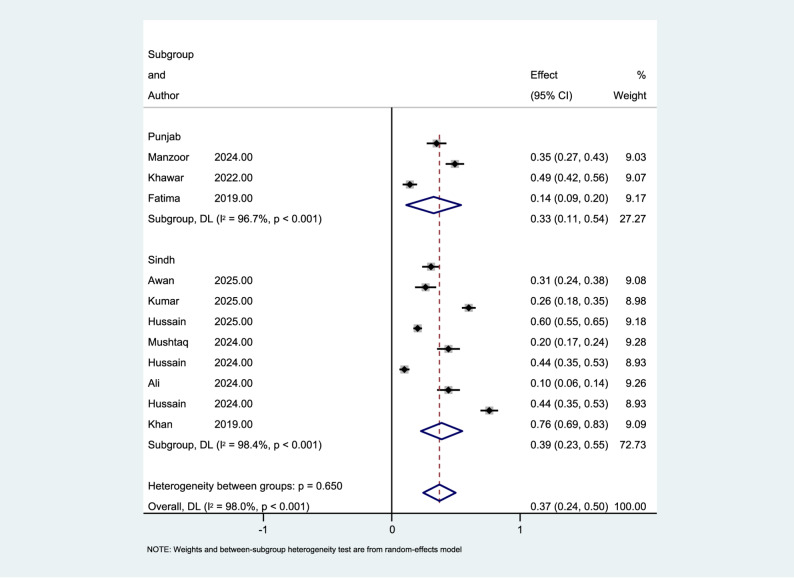
Forest plot of subgroup analysis by province (Punjab vs. Sindh)

However, the subgroup difference test indicated that the variation between the two provinces was not statistically significant (Q_between = 0.21, df = 1, *p* = 0.650). Therefore, while the reported prevalence in Sindh studies was slightly higher than in Punjab, this difference was not statistically significant. Substantial heterogeneity was observed within both subgroups, with I² = 96.7% for Punjab and I² = 98.4% for Sindh, indicating significant variability among the included studies in each subgroup. The subgroup forest plot in Fig. 4 compares the combined prevalence of depression in studies from Punjab and Sindh.

### Meta-regression analysis

**Fig. 5:**
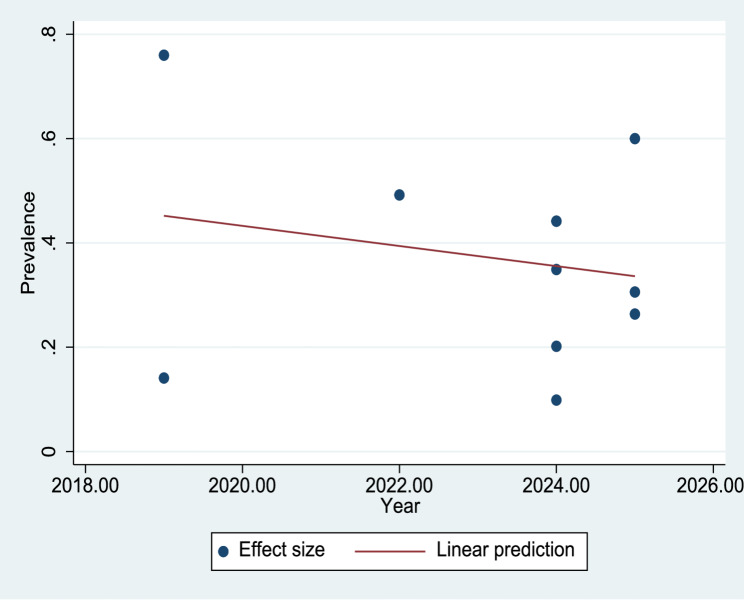
Meta-regression by year of study

A meta-regression analysis was conducted to examine the relationship between the year of study and the reported prevalence of depression among nursing students. The regression coefficient was β = −0.019, and the p-value was 0.528, indicating that the publication year was not a significant predictor of depression prevalence. The between-study variance was estimated at τ² = 0.041, and the residual heterogeneity remained high, with a residual I² value of 98.19%, suggesting considerable unexplained variability among the studies. Figure 5 illustrates that the publication year did not show a statistically significant association with the prevalence of depression among nursing students.

### Publication bias

**Fig. 6:**
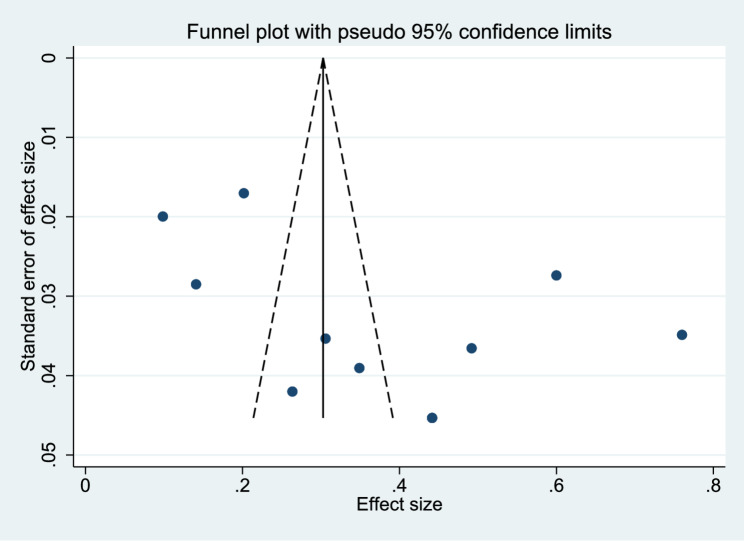
Funnel plot assessing publication bias among the included studies

Publication bias was assessed using Egger’s regression test and the Begg–Mazumdar rank correlation test. Egger’s test yielded a bias coefficient of 11.54 (*p* = 0.074), with a slope coefficient of − 0.01 (*p* = 0.938). Similarly, the Begg–Mazumdar test showed a Kendall’s tau of 0.22 (*p* = 0.389). Taken together, these results did not indicate statistically significant evidence of publication bias, as neither test demonstrated significant asymmetry in the distribution of effect sizes. As shown in Fig. 6, the funnel plot provides a visual assessment of potential publication bias. However, given the relatively small number of included studies, the statistical power of these tests is limited, and the presence of publication bias cannot be conclusively ruled out; therefore, the results should be interpreted with caution.

## Discussion

The findings of this systematic review and meta-analysis indicate that depression is a significant mental health concern among nursing students in Pakistan, with a pooled prevalence of 37%. This relatively high rate is consistent with global evidence demonstrating that students in health-related academic programs are particularly vulnerable to psychological distress [6]. However, the results of the included studies suggest that the manifestation of depression among nursing students in Pakistan is not uniform and is influenced by multiple contextual and individual factors. For instance, Ali et al. reported a lower prevalence of depression but higher levels of perceived stress, highlighting the substantial impact of academic workload and clinical training environments [23]. In contrast, Mushtaq and Ahmad identified perceived stress as a strong predictor of depressive symptoms, emphasizing the importance of developing coping strategies and stress management interventions to mitigate depression risk [21]. Moreover, Manzoor et al. demonstrated that a family history of psychological disorders may serve as an independent risk factor for depression, even when demographic variables do not show significant associations [24]. The review of the studies also pointed out how broader sociocultural factors can impact the mental health of nursing students. Some studies mentioned dissatisfaction with choosing nursing as a major, pressure from family to pursue fields with higher social status like medicine, and feelings of inferiority within the hierarchy between doctors and nurses. These factors can harm students’ self-esteem, motivation, and psychological well-being. When added to academic and clinical stress, these sociocultural influences may lead to lower academic satisfaction and a higher likelihood of depression among nursing students [19, 27]. It should also be noted that the inclusion of nursing students from different educational levels, including diploma, undergraduate, and postgraduate programs, may have contributed to the heterogeneity observed in the pooled prevalence estimates. These groups are likely exposed to distinct academic demands, clinical responsibilities, and professional expectations, which can differentially affect their psychological well-being. Diploma nursing students often encounter earlier and more intensive clinical exposure, while undergraduate and postgraduate students may experience greater academic pressure, research-related demands, and role-related expectations. Such variation in academic and clinical stressors may partially explain the differences in depression prevalence reported across the included studies.

One of the notable findings of this review is the limited geographical distribution of the included studies. Among the 11 studies analyzed, eight were conducted in the Sindh province and three in Punjab, while no research was identified from other major regions such as Khyber Pakhtunkhwa, Baluchistan, Gilgit–Baltistan, or Azad Kashmir. This lack of geographical representation may reflect disparities in research infrastructure, institutional research culture, and the level of attention given to mental health within nursing education programs across provinces. Consequently, the reported prevalence may not accurately represent the national situation, and it is possible that the prevalence of depression among nursing students in other regions is either lower or higher than the estimates presented here.

The meta-regression analysis indicated that the year of publication was not significantly associated with the reported prevalence of depression, suggesting that no clear increasing or decreasing trend was observed over time among nursing students in Pakistan. This finding indicates relative stability in reported prevalence across the included studies.

Additionally, the review revealed that research focusing on the prevalence of depression among nursing students in Pakistan has largely emerged after 2019, with no earlier studies specifically reporting prevalence rates in this population. This trend may reflect a growing academic and institutional recognition of mental health concerns among nursing students. At the same time, it underscores the lack of sufficient data to evaluate long-term patterns in this population.

Therefore, future research should adopt longitudinal designs and focus on the lived experiences of nursing students during periods of heightened stress, such as public health emergencies. Greater emphasis should also be placed on examining coping strategies, social support mechanisms, and educational structures to provide a more comprehensive understanding of the determinants of depression in this population Taken together, the high prevalence of depressive symptoms, along with recurrent reports of academic, clinical, and sociocultural stressors in the included studies, underscores the need for early identification strategies and supportive interventions within nursing education settings. In Pakistan, several initiatives aimed at improving student mental health have already been recommended by the Higher Education Commission (HEC) [29]. These include the establishment of university-based counseling and student wellbeing services [30], as well as the implementation of structured mentorship programs to strengthen communication and support between faculty members and students. Such initiatives may represent feasible and contextually appropriate strategies to support the mental wellbeing of nursing students. Another limitation relates to the exclusion of studies that used instruments combining depression with other psychological constructs, such as the Aga Khan Stress and Depression Scale. This scale reports depression together with stress, which does not allow for the independent estimation of depression prevalence. To maintain methodological consistency and comparability across studies, such studies were excluded from the meta-analysis. However, this decision may have resulted in the exclusion of some potentially relevant studies and could have influenced the overall pooled prevalence estimate.

Another important consideration is the quality and source of publication of the studies included. Only one study was found through the Medline database, while the other ten studies were published in local or regional scholarly journals primarily indexed in Google Scholar. Some of these journals may not follow strict peer-review processes or adhere to international methodological standards, which could impact the reliability and accuracy of the pooled prevalence estimates. While statistical tests for publication bias (Egger’s and Begg’s tests) did not show significant bias, the lack of high-quality studies in major international databases leaves open the potential for reporting or publication bias.

### Limitations

This study has several important limitations that should be considered when interpreting the findings. First, many of the included studies did not report key contextual variables, such as the type of institution (public or private), year of data collection, or gender-specific prevalence, which limited our ability to conduct subgroup and moderator analyses. The absence of these variables may have masked meaningful sources of variability and contributed to the extremely high heterogeneity observed, potentially leading to an over- or underestimation of the pooled prevalence.

Second, although a comprehensive search was conducted across major international databases and local peer-reviewed journals, grey literature (including theses, institutional reports, and unpublished studies) was not systematically searched. This may have resulted in the omission of studies with null or lower prevalence estimates, thereby introducing a degree of selection bias and influencing the magnitude of the pooled estimate.

Third, the geographical representation of the included studies was limited. All eligible studies were conducted only in the provinces of Sindh and Punjab, while no studies meeting the inclusion criteria were identified from other provinces such as Khyber Pakhtunkhwa or Balochistan. Therefore, the findings may not fully represent the prevalence of depression among nursing students across all regions of Pakistan.

Fourth, although provincial subgroup analyses were performed, the extremely high within-group heterogeneity (I² > 96%), wide confidence intervals, and the limited number of studies within each province substantially reduce the reliability and interpretability of the subgroup pooled prevalence estimates.

Finally, the substantial heterogeneity across studies limits the quantitative interpretability and generalizability of the pooled prevalence. As such, the summary estimate should be interpreted as an approximate measure reflecting an average across diverse study contexts rather than a precise national prevalence, and conclusions should be drawn with caution.

## Conclusion

In conclusion, this study represents the first systematic review and meta-analysis to examine the prevalence of depressive symptoms among nursing students in Pakistan. The findings suggest that depressive symptoms constitute an important mental health concern in this population; however, due to the substantial heterogeneity among the included studies and the limited geographical coverage, the pooled prevalence estimate should be interpreted with caution and viewed as a suggestive rather than a definitive estimate. Given the demanding academic and clinical nature of nursing education, these findings point to the need for practical and feasible interventions, including regular mental health screening of nursing students, the availability of accessible and confidential counseling services, and the integration of structured stress-management and coping-skills programs within nursing education settings. In the context of Pakistan, several initiatives related to student mental health have already been recommended by the Higher Education Commission (HEC). These include the establishment of counseling and student wellbeing services within universities, as well as the implementation of structured mentorship and mentee programs to strengthen communication and support between faculty members and students. Such initiatives may provide practical and culturally appropriate mechanisms to support the psychological wellbeing of nursing students and facilitate early identification of mental health concerns .Future research should involve broader geographical representation and more detailed reporting of demographic and institutional characteristics to generate more precise and generalizable estimates and to better inform targeted mental health interventions for nursing students in Pakistan.

## Supplementary Information

Supplementary Material 1.

## Data Availability

No datasets were generated or analyzed during the current study.
