# Supplementary material for: Depression among nursing students in Pakistan: a systematic review and meta-analysis
Source: BMC Psychol. 2026 Apr 14;14:768. doi: 10.1186/s40359-026-04385-w (PMC13188749; doi:10.1186/s40359-026-04385-w)
Supplement: Supplementary file 1 — Supplementary Material 1. [file 40359_2026_4385_MOESM1_ESM.docx]

**Search Strategy**

Search Date: 10 Oct 2025

**1. Ovid MEDLINE(R) ALL**

| 1 | exp Depression/ or exp Depressive Disorder/ or depress*.ti,ab,kf. | 692886 |
| --- | --- | --- |
| 2 | Students, Nursing/ or exp Education, Nursing/ or Schools, Nursing/ or (nurs* adj5 (student* or trainee* or learner* or education or training or school*)).ti,ab,kf. | 153436 |
| 3 | exp Pakistan/ or Pakistan*.ti,ab,kf. | 43938 |
| 4 | 1 and 2 and 3 | **9** |

**2. Embase 1974 to 2025 Week 40 (Ovid)**

| 1 | exp depression/ or depress*.ti,ab,kf. | 1132511 |
| --- | --- | --- |
| 2 | exp nursing student/ or exp nursing education/ or (nurs* adj5 (student* or trainee* or learner* or education or training or school*)).ti,ab,kf. | 159690 |
| 3 | exp Pakistan/ or exp Pakistani/ or Pakistan*.ti,ab,kf. | 62033 |
| 4 | 1 and 2 and 3 | **13** |

**3. APA PsycInfo 1967 to October 2025 Week 1 (Ovid)**

| 1 | exp "depression (emotion)"/ or depress*.ti,ab,hw. | 393741 |
| --- | --- | --- |
| 2 | Nursing Students/ or Nursing Education/ or (nurs* adj5 (student* or trainee* or learner* or education or training or school*)).ti,ab,hw. | 29119 |
| 3 | Pakistan*.mp. | 6360 |
| 4 | 1 and 2 and 3 | **2** |

**4.** **CINAHL with Full Text (Ebscohost)**

| S1 | (MH Depression+) OR depress* | 248,378 |
| --- | --- | --- |
| S2 | (MH "Students, Nursing+") OR (MH "Education, Nursing+") OR (MH "Schools, Nursing") OR (nurs* N5 (student* OR trainee* OR learner* OR education OR training OR school*)) | 191,220 |
| S3 | (MH Pakistan) OR (MH Pakistanis) OR Pakistan* | 9,674 |
| S4 | S1 AND S2 AND S3 | **5** |

**5. Scopus**

| 1 | TITLE-ABS-KEY (depress*) | 1,226,958 |
| --- | --- | --- |
| 2 | TITLE-ABS-KEY(nurs* W/5 (student* OR trainee* OR learner* OR education OR training OR school*)) | 194,219 |
| 3 | TITLE-ABS-KEY (Pakistan*) | 133,161 |
| 4 | #1 AND #2 AND #3 | **14** |

**6. Web of Science Core Collection (SCIE, SSCI, and ESCI)**

| 1 | TS= depress* | 960,294 |
| --- | --- | --- |
| 2 | TS= (nurs* NEAR/5 (student* OR trainee* OR learner* OR education OR training OR school* )) | 83,651 |
| 3 | TS= Pakistan* | 93,212 |
| 4 | #1 AND #2 AND #3 | **18** |

| 1 | Medline | 9 |
| --- | --- | --- |
| 2 | Embase | 13 |
| 3 | APA PsycInfo | 2 |
| 4 | CINAHL with Full Text | 5 |
| 5 | Scopus | 14 |
| 6 | Web of Science Core Collection | 18 |
| **Total** | | **61** |

Total records retrieved: 61

Duplicate records removed: 28

Records remaining for screening: 33
